# A scoping review of the evidence for the impact of pharmacological and non-pharmacological interventions on shift work related sleep disturbance in an occupational setting

**DOI:** 10.12688/wellcomeopenres.17002.1

**Published:** 2021-08-03

**Authors:** Rebecca Conway-Jones, Ella Dunlop, Simon Kyle, David Ray, Nia Roberts, Andrew Farmer

**Affiliations:** 1 Medical Science Division, University of Oxford, Oxford, UK; 2 Nuffield Department of Clinical Neurosciences, University of Oxford, Oxford, UK; 3 NIHR Oxford Biomedical Research Centre, John Radcliffe Hospital, Oxford, UK; 4 Oxford Centre for Diabetes, Endocrinology and Metabolism, Oxford, UK; 5 The Bodleian Libraries, University of Oxford, Oxford, UK; 6 Nuffield Department of Primary Care Health Sciences, University of Oxford, Oxford, UK

**Keywords:** Shift work disorder, shift work related sleep disturbance, occupational health, shift work, armodafinil, modafinil, hypnotics

## Abstract

**Background:** Shift work is essential in society but can be detrimental to health and quality of life and is associated with decreased productivity and increased risk of accidents. Interventions to reduce these consequences are needed, but the extent and range of trial evidence for interventions for those most affected by their shift-work schedules is unclear. We therefore carried out a scoping review to assess the availability of evidence to inform the development and evaluation of future interventions.

**Methods:** We aimed to identify clinical trials of any intervention for shift work-related sleep disturbance that included a comparator group, where the intervention was delivered in an occupational setting. We searched Cochrane Central Register of Controlled Trials, Cochrane Database of Systematic Reviews, CINAHL, EMBASE, Medline and Science Citation Index from inception to 30 ^th^ March 2020 for relevant citations. Citations were screened by two independent reviewers, a third reviewer resolved disagreements. Data were extracted by two independent reviewers.

**Results:** From 1250 unique citations, 14 studies met inclusion criteria for comparative trials of treatment in an occupational setting. There were five trials of hypnotics, five trials of stimulants, and four trials of non-pharmacological therapies (cognitive behavioural therapy, light therapy, aromatherapy and herbal medicine). Outcomes included sleep parameters, day-time sleepiness, and quality of life. There were no consistently reported outcomes across trials.

**Conclusions:** Interventions fell into three distinct groups investigated in distinct time periods without progression from efficacy trials to wider-scale interventions. The lack of consistent patient-reported outcome measures limits synthesising findings. Some trials focussed on optimising sleep, others on reducing wake-time sleepiness. Adequately powered trials of existing interventions are needed, with the development and testing of novel combination treatments in patients with well-defined shift work sleep disorder. A core set of clinically relevant outcomes will develop and standardise the evidence-base for shift work sleep disorder.

## Introduction

Shift work is increasing and often unavoidable in modern society. In 2015, 21% of the European Union working population carried out shift work ^1^. For individuals, shift work has been linked to reduced quality of life, increased risk of workplace accidents ^2, 3^, sleep loss, obesity, type 2 diabetes, coronary heart disease, some cancers ^4^ and depression ^5^. In an occupational setting this has been associated with lost productivity and increased errors ^3^. This is of particular concern where work performance is related to health and safety, for example in healthcare, emergency response and mining industries.

Shift work sleep disorder (SWD) refers to the sleep disturbance experienced by a subset of shift workers who respond particularly poorly to their shift work schedule ^6^. SWD is chiefly defined as having insomnia and/or excessive sleepiness temporally associated with shift work lasting ≥3 months, and where the sleep disturbance is not better explained by another diagnosis ^7^. A diagnosis of SWD is associated with a greater risk of poor quality sleep, subjective health complaints and poor coping ^8^, peptic ulcers, sleepiness-related accidents, absenteeism and depression when compared to shift workers without SWD ^2^. SWD represents an area of therapeutic interest for individuals and institutions alike. However, the range of and evidence for interventions in mitigating associated impairments is unclear.

Existing reviews of interventions to improve sleep, sleepiness and related outcomes for shift workers found low quality evidence for some interventions ^9, 10^. Melatonin, armodafinil, modafinil, caffeine and naps were all found to have low-quality evidence for their efficacy in improving one or more outcome domains ^9^. Another review found studies of bright light, napping, physical exercise and sleep education as interventions for shift-workers, but concluded there was too much uncertainty to determine their impact ^10^. To our knowledge, there has been no systematic review of clinical trials encompassing the full range of interventions focusing on shift-work related sleep disruption.

Here we present a scoping review of the available evidence from comparative studies examining the impact of interventions for SWD. We set out to identify all trials of shift workers with SWD, or sleep disturbance likely to be SWD that had a comparator group. We sought to establish the types and extent of available outcome data on this topic; how this has been reported and whether an informative quantitative data synthesis would be possible. 

## Methods

### Study design

We carried out a scoping review to identify the main sources and extent of evidence available in the published literature ^11^.

### Eligibility criteria

***Type of trials.*** We included randomised controlled trials (RCTs), randomised crossover trials and parallel group trials.

***Population.*** We included trials carried out with workers who were undertaking shift work and who had SWD as defined by the International Classification of Sleep Disorders (ICSD) criteria at the time when the trial in question was carried out. We also included studies where shift-workers were selected for having some level of sleep disturbance, that was not better explained by a known, non-SWD diagnosis, such as obstructive sleep apnoea (OSA) or narcolepsy. We excluded trials conducted on shift-workers unselected for having sleep problems, or conducted solely on shift-workers selected for having OSA or narcolepsy. We excluded studies in which airline cabin-crew or military personnel were the primary population group, as we considered the aetiology of SWD alongside frequent crossing of time zones likely to be different to that of SWD in the general population ^9^.

We included only trials where shift workers received a study treatment in an occupational setting. We included trials where final outcomes were measured in a simulated shift work environment, if participants had been undergoing the treatment in a real shift-work environment previously. For example, where participants had taken modafinil or armodafinil before shifts for a number of months, and then completed a study night in a laboratory setting ^12^.

***Interventions.*** We included trials with any intervention, or combination of interventions, aimed at preventing or reducing the effects of SWD on sleepiness when awake, sleep disturbance, and associated functional impairment (e.g. reduction in wellbeing, depressive symptoms). We categorised interventions into pharmacological hypnotics, pharmacological stimulants, and non-pharmacological therapies. We included trials where interventions were compared to placebo, ‘usual care’, no intervention or to each other. Example interventions for pharmacological stimulants might include modafinil or armodafinil. Example interventions for pharmacological hypnotics might include melatonin or zopiclone.

***Outcomes.***

We set out to identify and record when studies had used the following types of outcome measures.

1.    Sleep-wake outcomes:
     a.   Sleep outcomes: Measures of sleep parameters, such as total sleep time (TST), sleep efficiency (SE), sleep onset latency (SOL) or wake after sleep onset (WASO), or other measures of sleep quality such as number of awakenings.
     b.   Wake-time outcomes: Measures of alertness or sleepiness during waking hours, including Karolinska, Epworth or Stanford sleepiness scales (KSS, ESS, SSS); multiple sleep latency tests (MSLT).
     c.   Combined sleep-wake outcomes: Comprising both wake-time and sleep-time components, for example the Insomnia Severity Index (ISI) or Pittsburgh Sleep Quality Index (PSQI)
2.    Measures of impairment, including global measures of health-related quality of life, daytime functioning, and depressive symptoms.

We did not define the instrument of measurement to be used for any given parameter.

We also planned to record when studies reported outcomes of:

• Adverse events
• Injuries or accidents whilst at work or commuting.

### Search method

Electronic searches were conducted for Cochrane Central Register of Controlled Trials, Cochrane Database of Systematic Reviews, CINAHL, EMBASE, Medline and Science Citation Index from inception to 30 ^th^ March 2020. An example search strategy is shown in Table 1. The same search terms were used for all databases. We did not restrict by language. Title and abstract of all citations identified using this search strategy were screened for exclusion by two independent reviewers (R.C-J., E.D.) using Rayyan software ^13^. Conflicts were resolved with a third reviewer (A.F.). The full text of the remaining citations was then reviewed. Citations were only included in the study where they referred to a published journal article.

**Table 1.** Search Strategy Used for Medline.

1	shift work schedule/ or work schedule tolerance/
2	((shift* or night*) adj3 work*).ti,ab,kw.
3	((night* or rotat* or late) adj3 shift*).ti,ab,kw.
4	1 or 2 or 3
5	exp Sleep Wake Disorders/
6	(sleep* adj3 (disorder? or disturb* or problem?)). ti,ab,kw.
7	sleeplessness.ti,ab,kw.
8	(insomnia? or dysomnia? or parasomnia?).ti,ab,kw.
9	5 or 6 or 7 or 8
10	4 and 9
11	limit 10 to ("systematic review" or systematic reviews as topic or "reviews (maximizes specificity)")
12	randomized controlled trial.pt.
13	controlled clinical trial.pt.
14	randomized.ab.
15	placebo.ab.
16	drug therapy.fs.
17	randomly.ab.
18	trial.ab.
19	groups.ab.
20	(crossover or cross over).ti,ab.
21	(quasi* adj2 random*).ti,ab.
22	12 or 13 or 14 or 15 or 16 or 17 or 18 or 19 or 20 or 21
23	exp animals/ not humans.sh.
24	22 not 23
25	10 and 24
26	11 or 25

### Data extraction, analysis and synthesis

Data were extracted manually by two independent reviewers using a custom spreadsheet [Microsoft Excel, Version 16.37, Copyright 2020]. We extracted study characteristics. We aimed to extract data about all outcomes relevant to this review to establish the extent of available data. Where studies reported multiple measures in the same domain (for example, multiple measures of quality of life), we selected externally validated measures.

## Results

### Search results and study characteristics

**Figure 1.**
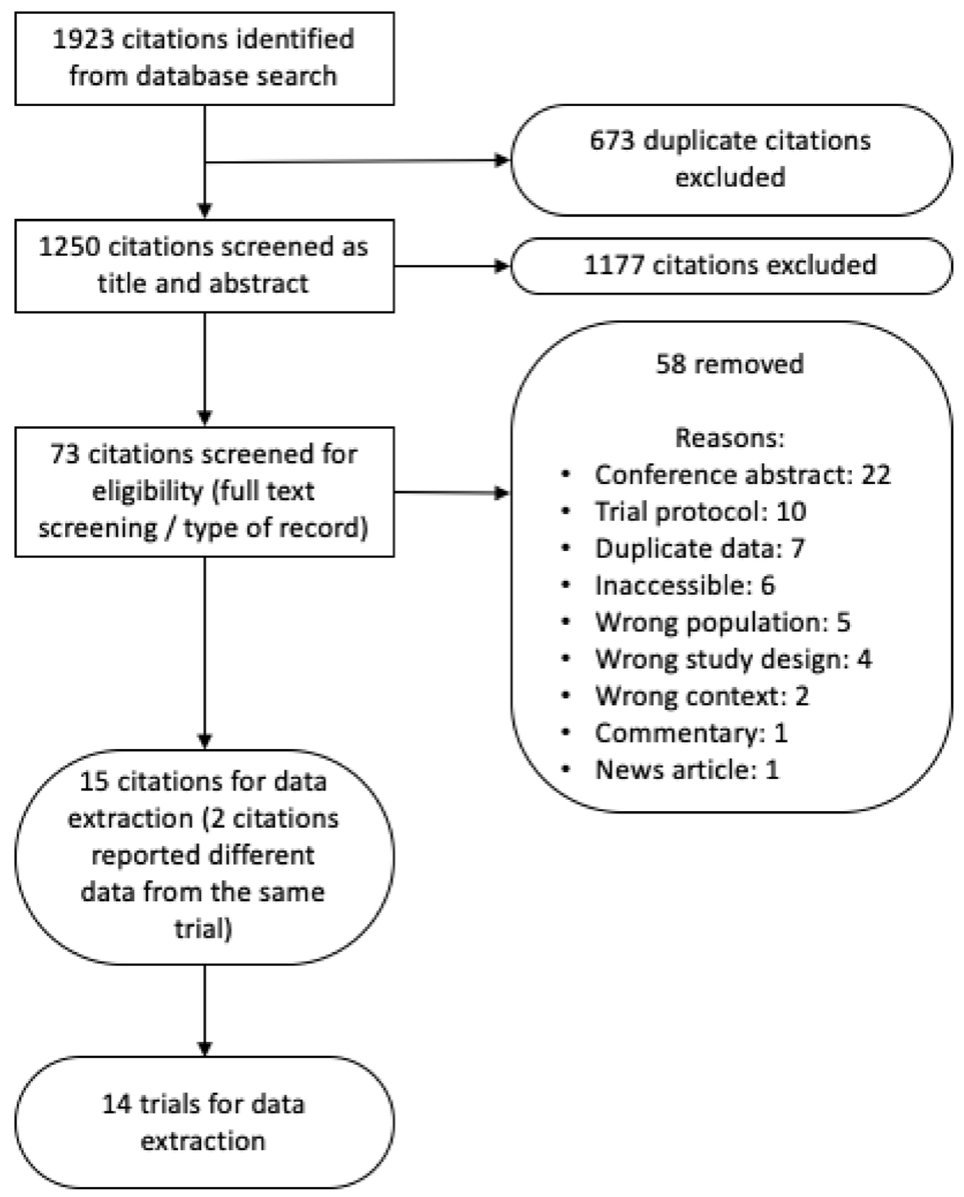
Preferred Reporting Items for Systematic Reviews and Meta-Analyses (PRISMA) flow diagram of study selection.

From 1250 unique citations, 14 trials met inclusion criteria for data extraction and analysis. Two included citations reported different outcomes from the same trial ^14, 15^. As each citation reported different outcome data the trial is referenced to both citations ^14, 15^. Figure 1 presents a Preferred Reporting Items for Systematic Reviews and Meta-Analyses (PRISMA) flow diagram showing reasons for study exclusions. In total, eight of the included trials were randomised controlled trials ^12, 14– 21^. The remainder were cross-over ^22– 24^ and a parallel group design ^25– 27^.

The studies were carried out over a wide geographical area, including Europe, Asia, and North America ( Table 2). There were a wide range of occupations evaluated including jobs in health care, industry, security services and broadcasting. Studies included those with rotating and fixed shifts. Five trials applied ICSD criteria for SWD in use at the time of the study; these were all studies of stimulants ^12, 14, 15, 20, 26, 27^. Interventions evaluated were hypnotic ^21– 25^ and stimulant drugs ^12, 14, 15, 20, 26, 27^, cognitive behaviour therapy ^17^, aromatherapy ^18^, light therapy ^19^ and Shimian granules (a form of a Chinese herbal medicine) ^16^. With one exception, the studies of hypnotics were carried out between 1989 and 2008, stimulants between 2005 and 2012 and the non-pharmacological interventions since 2013. Funding came from a variety of sources, although those studies evaluating stimulants were funded by pharmaceutical companies. All studies whose authors declared a potential conflict of interest with a relevant pharmaceutical company were studies of stimulants ^12, 14, 15, 20, 26, 27^.

**Table 2.** Characteristics of included studies.

	Study			Population						Intervention. Control was placebo unless otherwise stated	Design	Outcome focus	Timing			Funding Source	Conflict of interests with relevant pharmaceutical company declared?
	Author	Date	Country	Industry & Role	Type of shift work	Hours worked if reported	Criteria used for SWD or sleep disorder *	Sample size. Total completed (recruited and eligible)					Duration of intervention		Duration of study		
								Intervention	Control				Intervention	Control			
**Hypnotics**	Sadeghniiat- Haghighi, K	2016	Iran	Oil refinery	Rotating, 1 week day shift 1 week night shift.	N/A	Sleep disturbance assessed with ISI item 1a, PSQI item 2.	39 (50)		Melatonin 3mg OD	Randomised, double blind, placebo controlled crossover study	Sleep outcomes and other	3 nights, 2 week wash out		3 weeks	Tehran University of Medical Sciences and health services; Osvah Pharmaceutical Co	No
	Sadeghniiat- Haghighi, K	2008	Iran	Nursing	Unspecified.	N/A	Sleep problems reported in a baseline questionnaire	86 (118)		Melatonin 5mg OD	Randomised, double blind, placebo controlled crossover study	Sleep outcomes	1 night, 4 days wash out		<2 weeks	Tehran University of Medical Sciences	No
	Bozin- Juriacic, J	1996	Croatia	Security firm	Rotating, 7 night shifts, week rest, week of mornings and week of afternoons.	Night shifts were 22:00- 06:00	Self reported night work related insomnia.	9	9	Zopiclone 7.5mg OD	Parallel group	Sleep outcomes	1 week for each treatment, 3 week wash outs		12 weeks	May and Becker and Phone-Pulenc Santé	No
								11		Nitrazepam 5mg OD							
	Gigli	1993	Italy	Nursing	5 day rotation	Shifts: 7:00-14:00; 14:00-22:00; 22:00-7:00	Symptoms of insomnia considered by subjects or physicians to be clinically relevant.	13 (16)		Brotizolam 0.25mg OD	Double blind crossover study	Other	15 days with 15 day wash out		45 days	Boehringer Ingelheim Italia	No
	Monchesky	1989	Canada	Automobile plant	Rotating: Two weeks alternating	Shifts: 07:00- 15:30; 18:00- 02:30 Monday to Friday.	Insomnia for 3 or more consecutive night shifts via sleep latency, nightly awakenings, TST and poor sleep quality.	25 (25)	20 (25)	Zopiclone 7.5mg OD	RCT	Sleep outcomes	2 weeks		2 weeks	None declared	No
**Stimulants**	Erman, M K **	2011 & 2012	USA	Varied	Mixture. 93% permanent	≥5 nights per month, each 6-12 hours, with ≥6 hours between 22:00-08:00 and ≥3 consecutive nights	Excessive sleepiness late in shift associated with ICD2 SWD. Functionally impaired (GAF<70)	158 (184)	167 (187)	Armodafinil 150mg OD	RCT	Wake outcomes and AE	6 weeks		6 weeks	Cephalon Inc	Yes
	Tembe, D V	2011	India	Unspecified	Unspecified.		ICD criteria for SWSD	104 (105)	105 (106)	Armodafinil 150mg OD (Control: Modafinil 200mg OD)	RCT	Wake outcomes and AE	12 weeks		12 weeks	Emcure Pharmaceuticals Ltd.	Yes
	Czeisler, C A	2009	USA & Canada	Varied	Mixture. 86.5% permanent.		ICD2 criteria; <6 minutes on MSLT during a night shift; SE of 87.5% or less on daytime polysomnography	93 (127)	84 (127)	Armodafinil 150mg OD	Randomized, double-blind, placebo-controlled, parallel-group, multicenter study	Sleep outcomes, wake outcomes, other & AE	12 weeks		12 weeks	Cephalon Inc	Yes
	Erman, M K	2007	USA	Varied	Unspecified.		ICD	129 (185)	60 (93)	Modafinil 200mg or Modafinil 300mg OD	Randomised double blind placebo controlled parallel group trial	Other	12 weeks		12 weeks	Cephalon Inc	Yes
	Czeisler, C	2005	USA	Varied	Mixture. 90% permanent.		ICD2 criteria; <6 minutes on MSLT during a night shift; SE of 87.5% or less on daytime polysomnography	76 (96)	81 (108)	Modafinil 200mg OD	RCT	Sleep outcomes, wake outcomes, other & AE	12 weeks		12 weeks	Cephalon Inc	Yes
**Non-pharmacological**	Zhang, L	2020	China	Nursing	Forward rotating shift nurses	Shifts: 07:30-17:30; 12:30-22:30; 20:00-08:00. 5/7 days a week.	Score of ≥8 on the PSQI.	15 (19)	23 (25)	Shimian granules and sleep hygiene education (Control: sleep hygiene education only)	RCT	Other	1 month		1 month	Youth Science and Technology Innovation Personnel Training Project of Shaanxi Province; Special Support Scheme for ShaanxiProvince	No
	Jarnefelt, H	2019	Finland	Variety: health and social care, bakery, aviation	Varied	≥10% shifts beginning 07:00 or earlier, ending 22:00 or later or at least 3 hours of a shift falling between 23:00-06:00.	Non-organic insomnia; difficulty initiating or maintaining sleep for >30 min and use of sleep promoting medication at least 3 nights a week for at least 3 months;full time shift work	18 (30)	15 (24)	Group CBT-I (Control: Sleep hygiene education)	RCT	Sleep outcomes, other	Up to 10 weeks	1 session	6 months	Finnish Work Environment Fund; NordForsk, the Nordic Program on Health and Welfare	No
								16 (29)		Self help CBT-I (Control: sleep hygiene education)							
	Chang, Y Y.	2017	Taiwan	Nursing	Monthly rotating shifts.	N/A	A total PSQI ≥5	27 (27)	23 (23)	Aromatherapy massage	RCT	Other	4 weeks. 1 hour long treatment weekly.		5 weeks	Taichung Veterans General Hospital	No
	Huang, L B.	2013	Taiwan	Nursing	Rotating shifts	16:00-00:00; 00:00-08:00	ISI>14.	(46)	(46)	Bright light during nightshift + avoid daytime sun	RCT	Other	At least 10 days during 2 weeks.		At least 10 days during 2 weeks.	Chang Gung Memorial Hospital	No

Legend: Shift work disorder (SWD); insomnia severity index (ISI); Pittsburgh Sleep Quality Index (PSQI); once daily (OD); randomised controlled trial (RCT); total sleep time (TST); multiple sleep latency test (MSLT); International classification of disease (ICD); sleep efficiency (SE). *All patients being involved in shiftwork as defined by the study, and having no other conditions which better explain their sleep disturbance was an inclusion criterion so is not repeated here. **Different outcomes or timepoints from the same treatment population published separately.

The most commonly studied intervention was armodafinil (three trial populations) ^14, 15, 20, 26^. The other studies of stimulants used modafinil ^12, 27^. For hypnotics, there was variation in class, including studies of non-benzodiazepines (zopiclone ^21, 25^ and melatonin ^22, 24^), one study of a benzodiazepine (nitrazepam ^25^) and one study of a benzodiazepine analogue (brotizolam ^23^). No identical non-pharmacological intervention was studied by more than one study.

Four of the hypnotic intervention trials were conducted with rotating shift-workers ^21– 23, 25^. Studies of stimulants were on mixed populations of rotating and permanent shift-workers or did not specify their population characteristics in this way ^12, 14, 15, 20, 26, 27^. All included studies with a sample size >50 participants were studies of stimulants ^12, 14, 15, 20, 26, 27^. Non-pharmacological studies were on mixed populations of rotating and permanent shift-workers ^17^, or rotating shift workers ^16, 18, 19^.

### Reported outcomes

Table 3 shows the outcomes reported by the studies included in this review; the most commonly reported outcomes were TST and SOL, though they were measured by a variety of tools (polysomnography, actigraphy and sleep diaries).

**Table 3.** Selected Outcomes.

Study		Sleep-Wake Outcomes														Other outcomes				
		Sleep outcomes								Wake-time outcomes			Combined							
Author	Year	Measurement Tool (Sleep outcomes)	TST	SOL	SE	WASO	TBT	SQ	NoA	MSLT	KSS	SSS	ISI	PSQI	FOSQ10	SF-36/ RAND-36	RT	CGI-C	HADS	AE
Sadeghniiat- Haghighi, K	2016	Actigraphy	Y	Y	Y	Y							Y	Y						
Sadeghniiat- Haghighi, K	2008	Diary	Y	Y				Y	Y											
Bozin- Juriacic, J	1996	Diary	Y	Y	Y		Y	Y	Y											
Gigli	1993	-															Y			
Monchesky	1989	Diary	*	*				Y												Y
Erman, M K **	2011 & 2012	-									Y				Y			Y		Y
Tembe, D V	2011	-										Y								Y
Czeisler, C A	2009	Polysomnography	Y	Y	Y	Y			Y	Y	Y						Y	Y		Y
Erman, M K	2007	-													Y	Y				Y
Czeisler, C	2005	Polysomnography	Y	Y	Y		Y			Y	Y							Y		Y
Zhang, L	2020	-											Y	Y					Y	
Jarnefelt, H	2020	Actigraphy & Diary	Y	Y	Y	Y	Y						Y			Y				
Chang, Y Y.	2017	ECG based sleep detector	Y	Y	Y			Y						Y						
Huang, L B.	2013	-											Y						Y	

34 outcomes unlisted as not reported by more than one study. *Reported "speed of sleep onset" and "duration" of the sleep on a 0–9 score scale. ** Erman 2011 and 2012 placed together here as were conducted on the same study group Reported (Y); total Sleep Time (TST); Sleep onset latency (SOL); sleep efficiency (SE); wake after sleep onset (WASO); total bed time (TBT); sleep quality (SQ); number of awakenings (NoA); multiple sleep latency test (MSLT); epworth sleepiness scale (ESS); karolinska sleepiness scale (KSS); stanford sleepiness scale (SSS); insomnia severity index (ISI); Pittsburgh sleep quality index (PSQI); FOSQ10 (functional outcomes of sleep questionnaire 10); 36 item short form survey (SF-36/RAND-36); ; Simple reaction time (RT); Clinical global impression of change (CGI-C); hospital anxiety and depression scale (HADS); adverse events (AE).

***Sleep-wake outcomes.*** The most commonly used wake-time outcome was KSS. Studies of pharmacological interventions predominantly used MSLT and sleepiness scales ^12, 14, 20, 26^. No study used the ESS. Three studies of non-pharmacological interventions used the ISI ^16, 17, 19^. No non-pharmacological study used a sleepiness scale.

***Outcomes focused on other aspects of impairment.*** Clinical Global Impression of Change (CGI-C) score was used by three studies. Regarding depressive symptoms the Hospital Anxiety and Depression Scale (HADS) was used by two studies ^16, 19^. Two studies reported the 36-item Short Form Survey (SF-36) ^17, 27^, and two reported reaction time ^23, 26^.

***Adverse events and drop-outs.*** Adverse events were only reported by pharmacological studies. All studies of stimulants described monitoring for adverse events throughout the study periods. The protocols described varied but included combinations of subjective symptom reporting, physical examination and bedside and laboratory investigations. One study of zopiclone relied on study participants spontaneously reporting symptoms that they felt might be related to the study intervention ^21^. Few studies reported total counts of participants affected by any adverse events. Some, but not all, studies reported where drop-outs were due to adverse events. Only one study explicitly defined how adverse event severity was categorised ^12^ and one other stated only that event severity was determined by a site investigator ^26^.

## Discussion

Our scoping review provides evidence that a review to assess the effectiveness of interventions to treat SWD would not provide sufficient data for a comprehensive meta-analysis. There are too few studies amongst shift workers with SWD for any particular intervention or category of intervention. Even where there is more than one study, there are often methodological limitations preventing pooling of data using meta-analysis, such as different measurement tools for one outcome. Whilst we considered that a narrative review might be possible, the represented methodologies, interventions and data were so heterogenous it was not deemed appropriate at this point.

We did not critically appraise the included sources of evidence as this scoping review was conducted to provide an overview of the existing evidence regardless of methodological quality or risk of bias. However, we note that there were high rates of participant attrition in experimental and control arms in both pharmacological and non-pharmacological studies. Many drop-outs were unexplained.

Previous systematic reviews looking at a wider population of all shift-workers have similarly found a paucity of evidence regarding impact of interventions ^9, 10^. There are many interventions that have been considered in the literature for sleep disturbance ^28, 29^ that do not appear to have been evaluated in clinical trials with populations that meet the criteria for SWD, for example individual level interventions such as napping ^30^ and institutional level interventions such as optimising shift schedules ^31^. These interventions could be considered in future work looking at the SWD population specifically.

In this scoping review, we have grouped the short-term trial outcomes into sleep outcomes (ease of falling asleep, the continuity, duration and quality of obtained sleep), wake-time outcomes (sleepiness during waking hours), combined sleep-wake outcomes and other aspects of impairment. Sleep outcomes are the primary target of hypnotic pharmacological interventions whilst wake-time outcomes are that of wakefulness-promoting (stimulant) pharmacological agents. Non-pharmacological interventions vary in their putative mode of action.

The lack of consistent measurement of sleep outcomes was perhaps unsurprising then. Studies of the efficacy of stimulant pharmacological agents were aiming to promote wakefulness whilst at work and so often used real time state-based assessments like KSS and SSS. These were not used by any study of hypnotics or non-pharmacological interventions. Multiple studies reported a range of sleep outcomes (TST, SOL) but instrument of measurement was variable. Further work in this area could establish useful and replicable outcome measures for SWD.

The MSLT is described as the gold standard measure of sleepiness and is objective but many of the studies included in this review only used subjective measures of sleepiness ^14, 15, 20^. Whilst subjective measures of sleepiness are clinically relevant and sensitive to insufficient sleep acutely ^32^, they have a non-linear relationship to sleep debt and correlate poorly with sleep debt in the context of chronic sleep deprivation ^33^. Further, occupational impairment in SWD has been shown to be more strongly correlated to insomnia than to sleepiness so focusing on sleepiness may be unhelpful for work related outcomes ^34^. As such, it is likely a useful set of outcome measures for SWD will cover multiple domains of the disorder: insomnia symptoms, excessive waketime sleepiness and impact on functioning. These need to be consistent and standardised to prevent research waste where data from one study cannot be used to inform subsequent work or contribute to systematic reviews of the problem. We envisage shift-worker participation in future outcome measure development will be beneficial.

Advice for shift workers using available evidence has been described pragmatically elsewhere ^35^. There are also multiple guidelines available for clinicians treating patients with sleep disturbance due to shift work ^36, 37^. The evidence for the effectiveness of interventions currently available is unclear and the lack of follow-on evaluations from those identified in this review confirms the continuing uncertainty and gap in the evidence. Novel treatments and combinations of current treatment, targeting individuals where the type of shift work and the combination of symptoms experienced by an individual are needed.

## Data availability

All data underlying the results are available as part of the article and no additional source data are required.
