# Supplementary material for: A scoping review of the evidence for the impact of pharmacological and non-pharmacological interventions on shift work related sleep disturbance in an occupational setting
Source: Wellcome Open Res. 2023 Jan 6;6:198. Originally published 2021 Aug 3. [Version 2] doi: 10.12688/wellcomeopenres.17002.2 (PMC10280030; doi:10.12688/wellcomeopenres.17002.2)
Supplement: Peer review history

# Peer review: Reviewer response for version 2

**DOI:** 10.21956/wellcomeopenres.20689.r57934

**Published:** 2023-06-15

**Authors:** Ulises Jiménez Correa

**Affiliations:** 1 Universidad Nacional Autonoma de Mexico, Mexico City, Mexico City, Mexico

It is a very interesting scoping review with the aim to describe the evidence for the impact of pharmacological and non-pharmacological interventions on shift work disorder. Beyond interesting, I consider that it review is necessary. 

 I believe that the strength of this review consists in highlighting the little research that aims to determine the evidence of pharmacological/psychological treatment for sleep disorder due to work hours. The issue is very important because we live in a 24-hour society (i.e. factories, public security, mobility, hospital services), that require of workers for night shift and shift rotation; those who are at high risk of suffering symptoms of insomnia and daytime sleepiness; as well as metabolic alterations related to circadian rhythm disorders and accidents.

 Methods are according to introduction; results with the aim; and discussion with the results. However, with the aim of complementing the results section with the necessary information, I think that it is necessary to include an extra column in table 3 with the main findings of the included studies.

 I do not have any more comments.

Are the rationale for, and objectives of, the Systematic Review clearly stated?

Yes

Is the statistical analysis and its interpretation appropriate?

Not applicable

Are sufficient details of the methods and analysis provided to allow replication by others?

Yes

Are the conclusions drawn adequately supported by the results presented in the review?

Yes

Reviewer Expertise:

Sleep disorders

I confirm that I have read this submission and believe that I have an appropriate level of expertise to confirm that it is of an acceptable scientific standard.

# Peer review: Reviewer response for version 1

**DOI:** 10.21956/wellcomeopenres.18774.r51643

**Published:** 2022-08-05

**Authors:** Bobby Joseph

**Affiliations:** 1 Department of Community Health, St. John's Medical College, Bangalore, India

This being a scoping review, the authors have achieved their stated objectives of wanting to establish the types and extent of available outcome data on the impact of pharmacological and non-pharmacological interventions on shift work related sleep disturbance in the occupational setting.

 Having identified 1250 unique studies and whittling this down to just 14 trials that met the inclusion criteria, it was obviously a lengthy process. The findings of these 14 studies have been presented concisely both in the text as well as in the table. The paper has been well written and presents easy avenues for researchers who want to carry out their own research on the topic. If scoping reviews such as these fall within the purview of the journal, this article must surely Approved.

Are the rationale for, and objectives of, the Systematic Review clearly stated?

Yes

Is the statistical analysis and its interpretation appropriate?

Not applicable

Are sufficient details of the methods and analysis provided to allow replication by others?

Yes

Are the conclusions drawn adequately supported by the results presented in the review?

Yes

Reviewer Expertise:

Public health, primary health care, occupational health

I confirm that I have read this submission and believe that I have an appropriate level of expertise to confirm that it is of an acceptable scientific standard.

# Peer review: Reviewer response for version 1

**DOI:** 10.21956/wellcomeopenres.18774.r50051

**Published:** 2022-05-16

**Authors:** Kirstie N. Anderson

**Affiliations:** 1 Faculty of Medical Sciences, Newcastle University, Newcastle upon Tyne, UK; 2 Regional Sleep Service, Newcastle upon Tyne, UK

The paper is clear, well written and comes from expert colleagues. However, this is essentially stating that there is insufficient evidence to carry out meta-analysis - I agree with this and that there is very limited data. In fact there are no two trials using the same methodology or intervention. A minor point but I disagree with the opening sentence, there isn't really any evidence for shift work increasing, most work fewer hours with more protection of sleep periods in legislation compared to any period from the industrial revolution onwards. About 20% of people work some form of shift but this figure has been consistent over some time. Therefore many people work shifts, but always have. There isn't a clear definition of what a shift is or what makes a shift worker to help the non-expert.

 I had some questions about the interventions that were included and discussed. Brotizolam is a potent, old hypnotic no longer licensed for use in the UK, US or Canada which I think either needs to be clearly stated in the text or this study not used for those reasons. Likewise - there is a single PubMed reference only to shimian granules - a product that is not licensed and there is simply no information at all on what they are - a chinese complimentary medicine not in any way standard for sleep clinics. Again, there is a single RCT but nothing else at all in PubMed relevant for shimian granules.

 I appreciate the authors decided against critical review or narrative review but a small amount of extra information still seems necessary here.

 Accepting the timelines of any systematic review and inevitably - more studies will follow. However given such limited data - there is a very recent RCT using CBTi strategies just published ^1^. I think the paper ideally should be revised and this paper should be included, there is so little data that this would be a valuable addition, no stats were possible and all information presented is simply descriptive so I do not see this taking the authors significant time.

 There is also another drug company-listed trial with all results in clinicaltrials.gov I think it may have been reasonable to discuss this - Study of the Efficacy and Multiple-Dose Plasma Concentration-Time Profiles of Armodafinil and PROVIGIL - Study Results - ClinicalTrials.gov.

Are the rationale for, and objectives of, the Systematic Review clearly stated?

Yes

Is the statistical analysis and its interpretation appropriate?

Not applicable

Are sufficient details of the methods and analysis provided to allow replication by others?

Yes

Are the conclusions drawn adequately supported by the results presented in the review?

Partly

Reviewer Expertise:

Sleep medicine

I confirm that I have read this submission and believe that I have an appropriate level of expertise to confirm that it is of an acceptable scientific standard, however I have significant reservations, as outlined above.

## References

[1] : The effectiveness of an individualized sleep and shift work education and coaching program to manage shift work disorder in nurses: a randomized controlled trial. *J Clin Sleep Med* .2022;18(4) : 10.5664/jcsm.9782 1035-1045 10.5664/jcsm.9782 34870586PMC8974377

# Response

**Published:** 2022-11-29

**Authors:** Rebecca Conway-Jones

**Affiliations:** University of Oxford, UK

Dear Dr Anderson,

 Firstly, thank you for your considered and clear review. We appreciate the time and care which you took in reading this paper and making your comments. Our responses to the points raised are below. I have endeavoured to give line numbers for each change to make reviewing the changes easier but am aware formatting carried out by the editorial team might alter these:

 Revision 1

- “A minor point but I disagree with the opening sentence, there isn't really any evidence for shift work increasing, most work fewer hours with more protection of sleep periods in legislation compared to any period from the industrial revolution onwards. About 20% of people work some form of shift but this figure has been consistent over some time. Therefore many people work shifts, but always have."
- *Thank you for clarifying this – we have edited the first part of the introduction to reflect this.*

Revision 2

- “There isn't a clear definition of what a shift is or what makes a shift worker to help the non-expert.”
- *Apologies for this oversight. We have added in a definition of shift-work to the first paragraph of the introduction.*

Revision 3

- “I had some questions about the interventions that were included and discussed. Brotizolam is a potent, old hypnotic no longer licensed for use in the UK, US or Canada which I think either needs to be clearly stated in the text or this study not used for those reasons.”
- *This is a very useful point. We have chosen to keep the study included as it does otherwise fit the inclusion criteria we have previously stated. However, we have made it clear in the third paragraph of the Results section that it is not licensed in the UK, US or Canada. For both this and the point about Shimian Granules we have then highlighted this again in the first paragraph of Discussion.*

Revision 4

- “Likewise - there is a single PubMed reference only to shimian granules - a product that is not licensed and there is simply no information at all on what they are - a chinese complimentary medicine not in any way standard for sleep clinics. Again, there is a single RCT but nothing else at all in PubMed relevant for shimian granules. I appreciate the authors decided against critical review or narrative review but a small amount of extra information still seems necessary here.”
- *Yes, similarly we agree this is important and are grateful for your highlighting this. We have added a sentence to this effect in Results (second paragraph). For both this and the point about Brotizolam we have then highlighted this again in the first paragraph of the Discussion.*

Revision 5

- “Accepting the timelines of any systematic review and inevitably - more studies will follow. However given such limited data - there is a very recent RCT using CBTi strategies just published ^1^ . I think the paper ideally should be revised and this paper should be included, there is so little data that this would be a valuable addition, no stats were possible and all information presented is simply descriptive so I do not see this taking the authors significant time.”
- *Yes thank you so much for raising this. We agree this is a useful trial to highlight given the paucity of trials otherwise. We have simply included it and explained in the Results section how it has been added after peer review (first paragraph of Results). Whilst this isn’t perfect we agree that including it, even post-hoc, is in keeping with the goals of a scoping review. It is a valuable addition for completeness of this paper though it has not changed our conclusions*
- *The main edits pertaining to this are found in Figure 1, Tables 2 and 3 and within the Results section.*

Revision 6

- “There is also another drug company-listed trial with all results in clinicaltrials.gov I think it may have been reasonable to discuss this - Study of the Efficacy and Multiple-Dose Plasma Concentration-Time Profiles of Armodafinil and PROVIGIL - Study Results - ClinicalTrials.gov.”
- *Thank you for highlighting this. To acknowledge this study we have added a line in the Methods  under 'Search Method' in order to make reference to it. In contrast to the above (Booker et al 2022), we couldn’t fully include this study as our Methods included only trials published in journal articles. Therefore, we cannot exclude the possibility we excluded other drug company-listed trials with a similar profile to this one on the basis they had not been published. However, we hope that by making reference to it our review will still serve to make readers aware of this available data.*
